# Phylogeny of Wilms tumor?

**DOI:** 10.1016/j.ebiom.2016.06.044

**Published:** 2016-07-04

**Authors:** Adrian Charles, Ian A. Greer

**Affiliations:** Department of Pathology, Sidra Medical and Research Center, PO Box 26999, Doha, Qatar

The paper by Cresswell et al. (2016) addresses important issues in understanding the heterogeneous pathogenetic pathways in Wilms tumors and also practical questions of how sensitive and specific is a tumor biopsy for identifying important genetic changes. Histopathologists are used to morphological heterogeneity in Wilms tumors, and so underlying genetic heterogeneity may not be a surprise.

Wilms tumors are one of the more common pediatric solid tumors and have shown a marked improvement in mortality through refinements in therapy, but tumor relapse and therapy related life-long toxicity mean more tailored approaches are required. If therapy is going to be based on the tumor genetics, we need a clear understanding of the pathogenetic pathways and the likelihood of identifying key prognostic/diagnostic information in a tumor biopsy.

The pathogenetic differences between children and adults is hinted at by the relatively simple cytogenetics of many pediatric tumors compared to adult cancers, and confirmed in some pediatric tumors (e.g. rhabdoid) by the few changes identified by deep sequencing (Fruhwald et al., 2016). Knudson's two-hit hypothesis explains why some pediatric tumors have few steps (Knudson, 2001). Wilms tumors though appear genetically heterogeneous, with some tumors showing few changes and others showing more changes as the current paper demonstrates (Cresswell et al., 2016). Wilms tumors also appear to be driven by similar factors as normal embryo-fetal development, (Willis, 1950, Hohenstein et al., 2015). Recently the importance of noncoding RNA genetic pathways in some groups of Wilms tumors is being identified (Walz et al., 2015). This suggests complex pathogenetic pathway involving epigenetic changes (methylation, histone and chromosome structure and noncoding RNAs) with failure of differentiation and gene mutations are required (Hohenstein et al., 2015). Some genetic changes are not single events but multiple changes occurring synchronously (chromothripsis) are seen in some pediatric tumors (Chen et al., 2015).

Clonal progression in some tumor development is suggested by morphological progression (e.g., as the colonic adenoma-carcinoma pathway) and genetic progression (e.g., the Fearon-Vogelstein pathway Fearon and Vogelstein, 1990). Steps in adult tumor pathogenesis was suggested by Armitage and Doll 60 years ago (Armitage and Doll, 1954), with around 7 steps. The question then for pediatric tumors, is how do steps occur at such a young age and are there fewer steps? One point to note is that small round blue cell tumors of childhood tend to be rapidly proliferative and time should be measured by cell cycles, rather than months and years. Morphological evidence of clonal progression is less clear in most pediatric tumors, but Wilms tumors with their precursor lesions and focal anaplasia suggest progression.

Darwinian clonal evolution will mean the tumor contains genetically heterogenous populations, until some factor selects one group over another. The conceptual distinction between inherent tumor aggressiveness and therapy resistance and prognosis was addressed by Beckwith (Beckwith et al., 1996) who noted that blastemal predominant Wilms tumors pre-therapy are generally responsive to chemotherapy, but present with a higher stage. A resistant component may be a small proportion until selected for, and therefore a prognostic genetic change may not be identified by a small biopsy, and this is shown in the current paper (Cresswell et al., 2016).

Currently we are able to deeply sequence a tumor but we need to understand what the genetic changes mean. Some changes will be important drivers of the tumor pathogenesis, others may be important supportive factors (such as increased Insulin-like Growth Factor (IGF)2), others such as p53 and possibly 1q gain are associated with prognosis and response to therapy rather than pathogenesis, yet others may be passenger changes having no survival effect on the tumor or they may be of uncertain significance. Furthermore, epigenetic changes appear to be crucial in many pediatric tumors. Some of these changes (referred to in the current paper Cresswell et al., 2016) are seen in the surrounding “normal” kidney, which may be the environment which assists tumor development (e.g., the paracrine effect of IGF2) or reflect a subpopulation that has a pro-oncogenic change but has been induced to differentiate and lost the ability to progress to a tumor.

This paper by Cresswell et al. (2016) therefore demonstrates that Wilms tumors should be regarded as a complex, heterogeneous system, with related subpopulations of tumor cells co-existing. Tumor samples should also be considered as epidemiological samples of related populations, and the likelihood of detecting minor populations considered.

This paper (Cresswell et al., 2016) suggests future investigations. The routine collection of tumor samples pre and post therapy, at relapse and from metastases (and in time circulating tumor nucleic acids), particularly from poor responders, needs promotion. Newer techniques such as single molecule sequencing will allow smaller subpopulations to be detected and quantified. Highlighted also in the paper is the use of a growing range of bioinformatic tools to assist our understanding and handling of the large data, to refine and personalize management for these tumors. This paper now adds phylogeny to our understanding of the group of tumors called Wilms tumor (Cresswell et al., 2016).

## Disclosure

The author declared no conflicts of interest.

## References

[bb0005] Armitage P., Doll R. (1954). The age distribution of cancer and a multi-stage theory of carcinogenesis. Br. J. Cancer.

[bb0010] Beckwith J.B., Zuppan C.E., Browning N.G., Moksness J., Breslow N.E. (1996). Histological analysis of aggressiveness and responsiveness in Wilms' tumor. Med. Pediatr. Oncol..

[bb0015] Chen X., Pappo A., Dyer M.A. (2015). Pediatric solid tumor genomics and developmental pliancy. Oncogene.

[bb0020] Cresswell G.D., Apps J.R., Chagtai T. (2016). Intra-tumor genetic heterogeneity in Wilms tumor: clonal evolution and clinical implications. Oncogene.

[bb0025] Fearon E.R., Vogelstein B. (1990). A genetic model for colorectal tumorigenesis. Cell.

[bb0030] Fruhwald M.C., Biegel J.A., Bourdeaut F., Roberts C.W., Chi S.N. (2016). Atypical teratoid/rhabdoid tumors-current concepts, advances in biology, and potential future therapies. Neuro-Oncology.

[bb0035] Hohenstein P., Pritchard-Jones K., Charlton J. (2015). The yin and yang of kidney development and Wilms' tumors. Genes Dev..

[bb0040] Knudson A.G. (2001). Two genetic hits (more or less) to cancer. Nat. Rev. Cancer.

[bb0045] Walz A.L., Ooms A., Gadd S., Gerhard D.S., Smith M.A., Guidry Auvil J.M. (2015). Recurrent DGCR8, DROSHA, and SIX homeodomain mutations in favorable histology Wilms tumors. Cancer Cell.

[bb0050] Willis R.A. (1950). The borderland of embryology and pathology. Bull. N. Y. Acad. Med..

